# Salivary Interleukins as Non-Invasive Biomarkers for Psoriasis: Advances and Challenges in Diagnosis and Monitoring

**DOI:** 10.3390/medicina61071180

**Published:** 2025-06-29

**Authors:** Anna Sora, Tony Hangan, Sergiu Ioachim Chirila, Leonard Gurgas, Mihaela Botnarciuc, Lavinia Carmen Daba, Ana Maria Cretu, Ionut Burlacu, Mihaela Zamfirescu, Adina Petcu, Adrian Cosmin Rosca, Ramona Mihaela Stoicescu, Lucian Cristian Petcu

**Affiliations:** 1 Doctoral School of Medicine, Ovidius University of Constanta, 900470 Constanta, Romania; 2 Center for Research and Development of the Morphological and Genetic Studies of Malignant Pathology [CEDMOG], 900591 Constanta, Romania; 3 Faculty of Medicine, Ovidius University of Constanta, 900470 Constanta, Romania; 4 Blood Transfusions Unit, Emergency Clinical County Hospital Constanta, 900591 Constanta, Romania; 5 Department of Pathology, Clinical Service of Pathology, “Sf. Apostol Andrei” Emergency County Hospital, 900591 Constanta, Romania; 6 Faculty of Pharmacy, Ovidius University of Constanta, 900470 Constanta, Romania; 7 Faculty of Dental Medicine, Ovidius University of Constanta, 900470 Constanta, Romania

**Keywords:** salivary biomarkers, psoriasis, interleukins, cytokines

## Abstract

*Background and Objectives*: Psoriasis is a chronic immune-mediated inflammatory disease requiring reliable diagnostic and monitoring tools. Salivary interleukins have emerged as promising non-invasive biomarkers, reflecting systemic inflammation and offering practical advantages such as ease of collection and improved patient compliance. *Materials and Methods*: This review synthesizes the current evidence on key salivary cytokines—IL-1β, IL-6, TNF-α, and IL-17—in relation to psoriasis pathogenesis, diagnosis, and treatment monitoring. It also compares saliva to blood-based diagnostics, emphasizing benefits like cost-effectiveness and suitability for repeated sampling. Methodological challenges, including heterogeneity in collection protocols and limited longitudinal data, are critically examined. *Results*: Advances in biologic therapies have deepened the understanding of psoriasis immunopathogenesis, highlighting interleukins as central biomarkers. Recent findings identify IL-37 and IL-38 as novel regulatory cytokines with anti-inflammatory roles. While elevated serum TNF-α levels in psoriatic patients are well documented, some inconsistencies persist. Notably, saliva has proven to be a viable alternative diagnostic fluid, supporting large-scale screening and routine clinical monitoring. *Conclusions*: Salivary interleukins—particularly IL-1β, IL-6, TNF-α, and IL-17—represent valuable, non-invasive biomarkers for early detection, disease severity assessment, and therapeutic response monitoring in psoriasis. Standardizing saliva-based methods and conducting large-scale studies are essential next steps to support their integration into personalized clinical practice.

## 1. Introduction

Psoriasis is a common, chronic, and clinically complex dermatological disorder that has historically been regarded as a condition marked by epidermal hyperproliferation and accelerated turnover, accompanied by an activated mononuclear cell infiltrate in the underlying dermis. Recent advances in the understanding of psoriasis have highlighted the critical involvement of T lymphocytes in its pathogenesis. The therapeutic efficacy of emerging T cell-targeted immunotherapies, as well as traditional antipsoriatic agents such as methotrexate, corticosteroids, and cyclosporine A, further underscores the central role of the immune system in this disease [1].

Currently, psoriasis research is largely driven by the hypothesis that it is an immune-mediated disorder characterized by aberrant keratinocyte proliferation under the influence of T lymphocytes [2,3,4,5]. Autoimmune and inflammatory diseases are typically classified according to the predominance of Th1- or Th2-mediated immune responses. Psoriasis is associated with the overexpression of pro-inflammatory cytokines derived from Th1 cells, along with a relative underexpression of Th2 cytokines [6].

The role of cytokines in the pathogenesis of psoriasis remains a major focus of ongoing research. However, extrapolating cytokine functions observed in in vitro studies to the in vivo context of psoriasis suggests that the interactions among cytokines in the human system may be significantly more intricate than those demonstrated under experimental conditions [7].

It has become increasingly evident that interleukins play a pivotal role in the pathogenesis of inflammatory dermatologic disorders. In this review, we aimed to identify the interleukins involved in the pathogenesis of psoriasis, with a particular focus on salivary interleukins. We begin by presenting the major known interleukin families and their fundamental roles in the normal immune response. We then examine relevant preclinical and clinical data that support the pathogenic involvement of interleukins in psoriasis [7,8].

The primary objective of this review was to highlight the potential of salivary interleukins as a non-invasive, cost-effective, and patient-compliant alternative for monitoring individuals with psoriasis.

## 2. Materials and Methods

This review was undertaken to synthesize and contextualize the existing knowledge on the role of salivary interleukins as non-invasive biomarkers in the diagnosis and monitoring of psoriasis. Given the diversity of study designs, the heterogeneity of methodologies, and the evolving nature of this research area, a narrative approach was deemed most appropriate to provide a comprehensive overview, identify knowledge gaps, and highlight future research directions.

The review focused on studies investigating salivary interleukins in patients with psoriasis, published in peer-reviewed journals up to April 2025. Both clinical and preclinical studies were considered if they included original data on interleukin expression or levels in saliva and explored their potential diagnostic, prognostic, or monitoring value in psoriasis. Studies focusing on interleukins in other bodily fluids, or unrelated to psoriasis, were excluded. The scope included advances in analytical methods, clinical relevance, and practical considerations related to salivary biomarker use.

Inclusion criteria were original research articles, studies involving human subjects diagnosed with any clinical form of psoriasis, studies reporting on salivary interleukin levels, and articles published in English. Exclusion criteria were editorials or conference abstracts, studies that investigated interleukins exclusively in serum, tissue, or other biological matrices, and studies not clearly identifying psoriasis as a target condition. These criteria were established to ensure the review remained focused, clinically relevant, and methodologically sound.

Literature saturation was achieved when no new relevant studies or perspectives emerged from repeated database searches and cross-referencing. We are confident that the sources included provide a sufficiently comprehensive foundation for the current state of research on this topic.

The literature search was conducted using PubMed, Scopus, and Web of Science, with the following keywords: “psoriasis,” “saliva,” “interleukin,” “biomarker,” and “non-invasive.” Titles and abstracts were screened for relevance, followed by a full-text review of eligible studies. Data were extracted on study design, sample size, type of interleukins investigated, methods of detection, clinical associations, and diagnostic or prognostic value. The analysis was descriptive and thematic, aiming to identify patterns, methodological trends, and clinical implications. Discrepancies or limitations in the findings were noted to inform both the strengths and challenges in this research field.

## 3. Results

### 3.1. The Context of Psoriasis as a Chronic Inflammatory Disease

Psoriasis is a chronic, proliferative, immune-mediated inflammatory disorder, characterized by a relapsing–remitting clinical course, affecting approximately 2–3% of the global population. Psoriasis occurs equally in both males and females and has a bimodal age of peak occurrence: between 20 and 30 and between 60 and 70 [9]. A recent systematic review investigating the epidemiology of psoriasis reinforces the notion that psoriasis represents a prevalent chronic inflammatory disease within the general population. This conclusion is supported by the analysis of 46 studies assessing the prevalence of psoriasis, as well as 7 studies evaluating the incidence of the disease in population-based cohorts [10].

Various environmental factors are implicated in the initiation or exacerbation of psoriasis in genetically predisposed individuals. These triggers include mechanical trauma (Koebner’s phenomenon), infections, certain medications, smoking, alcohol consumption, and psychological stress. Among pharmacological agents, several drugs have been specifically associated with the worsening of psoriatic lesions, including antimalarials, bupropion, β-blockers, calcium channel blockers, captopril, and fluoxetine [11]. Scratching and other forms of physical trauma are recognized as potential triggers for the onset of psoriasis, a phenomenon referred to as Koebner’s phenomenon. This process involves the development of psoriatic lesions at sites of skin injury, highlighting the interplay between mechanical damage and the underlying immunological mechanisms of the disease [12].

Clinically, psoriasis most commonly presents as well-demarcated, erythematous plaques covered by adherent silvery scales, with a predilection for the scalp and the extensor surfaces of the limbs [13]. Plaque psoriasis represents the most prevalent clinical form of psoriasis. In recent years, significant progress has been made in understanding its complex pathogenesis, genetic background, associated comorbidities, and the development of targeted biologic therapies. Plaque psoriasis is frequently associated with a broad spectrum of comorbid conditions, including psoriatic arthritis, cardiometabolic disorders, and psychological disturbances such as depression, all of which contribute to the overall disease burden and impact patients’ quality of life [14].

However, the impact of psoriasis extends beyond cutaneous manifestations, as the disease can significantly affect patients’ physical, emotional, and social well-being. The degree of disability and quality-of-life impairment experienced by individuals with psoriasis has been shown to be comparable to that observed in other severe chronic conditions, including malignancies and cardiovascular diseases [13].

Advances in understanding the immunopathogenic mechanisms underlying psoriasis have enabled the identification of key molecular mediators involved in the psoriatic inflammatory cascade. The pathogenesis of psoriasis is characterized by a complex interplay between multiple cell types and inflammatory cytokines. Both the innate and adaptive immune systems play critical roles in driving the inflammatory processes underlying psoriatic lesions [15].

Consequently, therapeutic strategies have significantly evolved, particularly with the introduction of biologic agents specifically targeting these pro-inflammatory pathways, thus modifying the natural course of the disease [16].

Biologic therapies have demonstrated the capacity to modulate the immunological background of psoriasis, contributing not only to the control of cutaneous manifestations but also to the prevention of irreversible tissue damage and systemic complications associated with chronic inflammation. Nevertheless, despite the remarkable efficacy of biologics, several challenges persist regarding their long-term safety profile, particularly in patients with systemic comorbidities or in specific populations requiring special therapeutic considerations [16].

### 3.2. The Role of Biomarkers in Monitoring Psoriasis

Biomarkers are clinically significant as they enable the quantitative assessment of diagnosis, disease pathophysiology, severity, and treatment response [17]. These biomarkers may include genetic, soluble, cellular, or tissue-associated markers.

Biomarkers assist healthcare professionals in identifying individuals at risk for severe disease manifestations or those likely to develop comorbidities. Additionally, they aid in predicting responses to various therapeutic interventions, including biologics, and facilitate the customization of treatment strategies for psoriasis [18].

As a systemic condition, there is a pressing need to identify potential biomarkers for evaluating disease severity, predicting therapeutic outcomes, and assessing associations with various systemic comorbidities. Various genetic markers not only serve as indicators of disease pathogenesis but also play a role in predicting the development of psoriasis and psoriatic arthritis. Personalized medicine tailors therapeutic strategies to the specific needs of psoriasis patients, optimizing treatment outcomes based on insights derived from these biomarkers [19].

Recent advances in biologic therapies have significantly contributed to elucidating the complex immunopathogenic mechanisms involved in psoriasis, highlighting the pivotal roles of biomarkers [20].

#### Different Types of Biomarkers in Psoriasis

*Systemic inflammatory markers:* They have emerged as promising tools for evaluating disease severity and systemic involvement in psoriasis. These include hematological ratios derived from complete blood count parameters and acute-phase reactants [20]. A recent study aimed to evaluate the systemic inflammatory burden associated with psoriasis by analyzing a range of inflammatory biomarkers such as neutrophil-to-lymphocyte ratio, platelet-to-lymphocyte ratio, monocyte-to-lymphocyte ratio, systemic immune-inflammation index, systemic inflammation response index, C-reactive protein, and monocyte/HDL cholesterol ratio and examining their correlation with disease severity [21].

*Keratinocyte markers:* Keratin expression patterns in psoriasis are significantly altered due to abnormal differentiation and hyperproliferation. Monoclonal antibodies targeting keratins—particularly K6, K16, and K17—have proven useful in studying epidermal pathology in psoriasis https://pubmed.ncbi.nlm.nih.gov/7577575/ [22].

*CD immune markers:* Psoriasis features an exaggerated immune response, with the infiltration of CD4+ and CD8+ T cells, dendritic cells such as CD11c+, and other leukocytes. These markers reflect the immunological profile of active disease, and they are used to study immune infiltration in psoriatic lesions [23].

In recent years, specific skin fluorescence techniques have emerged as promising non-invasive diagnostic tools, enabling the express detection of psoriatic lesions based on their distinct biochemical and structural properties. Skin fluorescence photography detects alterations in endogenous fluorophores and tissue architecture, offering a real-time, non-invasive assessment of psoriatic lesions based on their optical properties [24].

Compared to salivary interleukins—which are non-invasive, easily collected, and reflect systemic immune activation—systemic inflammatory markers are widely accessible and cost-effective but lack disease specificity; keratinocyte and CD markers offer high cutaneous specificity and mechanistic insights but require invasive sampling and are less practical for routine monitoring, while skin autofluorescence provides a non-invasive visual assessment of epidermal changes, though it remains technically limited and less validated across clinical settings [25,26].

### 3.3. Interleukins in Psoriasis—General Overview

#### Immunological Mechanisms Involved in Psoriasis

Psoriasis is a chronic inflammatory disease characterized by the profound dysregulation of the cytokine network, involving multiple self-amplifying feedback loops that accelerate pathogenic pathways. In genetically or immunologically predisposed individuals, psoriatic inflammation may be triggered by a variety of exogenous factors, including environmental exposures such as air pollutants, UV radiation, and other external stimuli such as trauma, medications, infections, or vaccinations [27].

Pattern recognition receptors (PRRs), particularly Toll-like receptors (TLRs), appear to play a pivotal role as key mediators in recognizing and responding to these triggering stimuli [28]. Cutaneous injury leads to the release of damage-associated molecular patterns (DAMPs), including double-stranded RNA (dsRNA), single-stranded RNA (ssRNA), and DNA from damaged cells, which subsequently activate TLR signaling pathways in various cell types, notably keratinocytes (KCs) and dendritic cells (DCs) [29,30].

**Figure 1:**
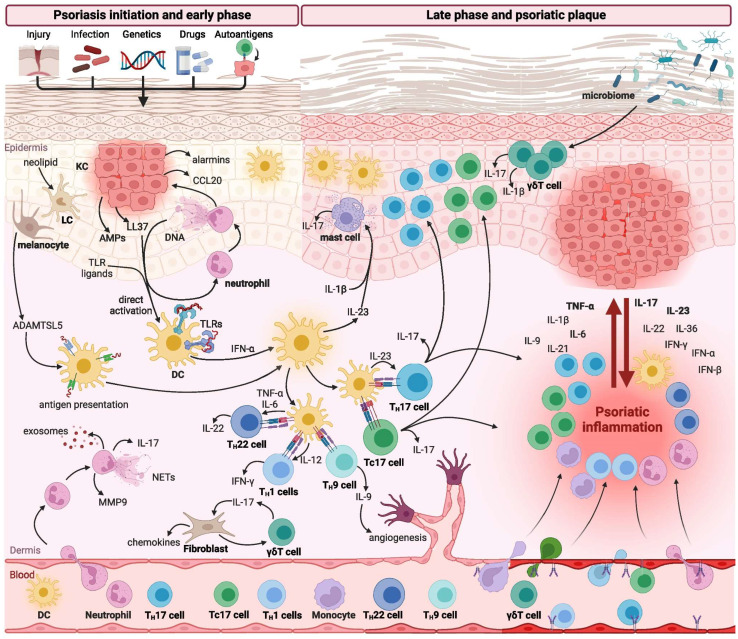
Immune response in psoriasis (reproduced from Izabela Sieminska et al., *Clinical Reviews in Allergy and Immunology*, published by Springer Nature, 2024) [26].

The activation of TLRs by DAMPs or pathogen-associated molecular patterns (PAMPs) induces the production of a broad spectrum of pro-inflammatory cytokines, thereby contributing to the initiation and amplification of the psoriatic inflammatory cascade [30,31] (Figure 1).

Various immune cell populations are critically involved in regulating the initiation, maintenance, and progression of psoriatic inflammation. Among these, T lymphocytes—particularly T helper 17 (TH17) cells—alongside dendritic cells (DCs), represent the central mediators driving the immunopathogenesis of psoriasis. In addition, several other immune cell subsets, including neutrophils, monocytes, macrophages, mast cells, and innate lymphoid cells (ILCs), have also been shown to contribute significantly to disease development and progression [25,26,32].

**Figure 2:**
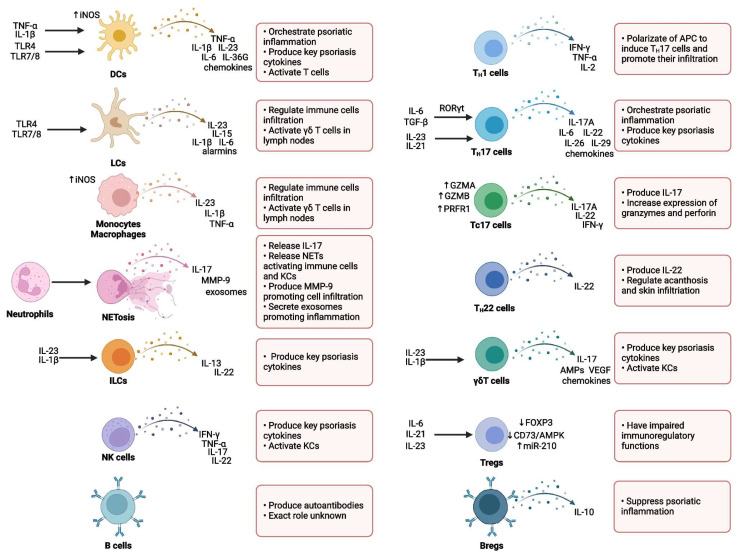
Immune cells in psoriasis (reproduced from Izabela Sieminska et al., *Clinical Reviews in Allergy and Immunology*, published by Springer Nature, 2024) [26].

Typically, innate immune cells, with neutrophils playing a predominant role, are essential in the early stages of psoriatic lesion formation. In contrast, the chronic phase of the disease, characterized by stable psoriatic plaques, is predominantly sustained by adaptive immune responses, particularly those mediated by T cells [33] (Figure 2).

### 3.4. Main Involved Interleukins 

Psoriasis is primarily characterized by dysregulated cytokine expression, which plays a pivotal role in the pathogenesis of the disease. Several approved therapies, as well as investigational treatments, employ monoclonal antibodies targeting specific cytokine proteins [15].

Cytokines are classified into distinct families, including interleukins—which are secreted molecules acting on leukocytes—the tumor necrosis factor (TNF) family, and chemokine proteins [34].

Numerous interleukins are actively involved in the pathogenesis of psoriasis, while certain interleukins may exert a protective effect by enhancing the expression of antimicrobial defense proteins [34].

In psoriasis, various stress-related triggers—ranging from environmental factors to genetic predispositions—can activate innate immune cells and keratinocytes to produce tumor necrosis factor-α (TNF-α), interferon-γ (IFN-γ), IFN-α, interleukin-6 (IL-6), and interleukin-1β (IL-1β) [35].

These cytokines subsequently activate dermal dendritic cells, which in turn secrete IL-12 and IL-23, promoting the differentiation of naive T cells into Th1 and Th17 subsets, respectively [32].

Th1 cells primarily produce TNF-α and IFN-γ, while Th17 cells secrete IL-17A-F and IL-22. These cytokines contribute to the activation and proliferation of keratinocytes, which further release a broad spectrum of pro-inflammatory cytokines and chemokines that drive the phenotypic features of psoriasis [35].

This inflammatory cascade operates through a positive feedback mechanism, facilitating the progression from disease initiation to its maintenance and chronicity [35].

The IL-23/IL-17 inflammatory cascade plays a central role in the pathogenesis of psoriasis. IL-23, synthesized by myeloid dendritic cells (mDCs), is present in high concentrations within psoriatic lesions and is essential for the maintenance of Th17 cells, which are a primary source of IL-17 [36]. While it was initially thought that IL-17 production was strictly dependent on IL-23, evidence suggests that IL-17 can also be produced independently of IL-23 through the innate immune system [37]. Beyond the well-characterized contribution of IL-17-producing T helper 17 (Th17) lymphocytes, recent evidence has emphasized the involvement of innate lymphoid cell type 3 (ILC3), which is capable of inducing psoriatic lesions independently of antigen-specific T cell responses. This mechanism is mediated by antimicrobial peptides released from activated keratinocytes and pro-inflammatory cytokines within the cutaneous microenvironment.

ILC3 cells are characterized by the nuclear expression of the retinoic acid receptor-related orphan receptor gamma t (RORγt), undergoing maturation under the influence of IL-7 and IL-23, and exerting their effects primarily through the secretion of IL-17 and IL-22. Notably, the increased numbers of ILC3 cells have been identified not only in the peripheral circulation and lesional skin of psoriatic patients but also within non-lesional skin areas, suggesting their broader involvement in disease pathogenesis [38].

Significantly elevated levels of IL-6 have been reported in active psoriatic plaques obtained from 35 patients with psoriasis, compared to biopsies from non-lesional skin. Moreover, increased IL-6 concentrations were also detected in the plasma of these patients, which may contribute to epidermal hyperplasia, given the proliferative effect of IL-6 on keratinocytes [39].

IL-8 levels are elevated both in psoriatic scale extracts and in suction blister fluids derived from psoriatic skin lesions [40]. Neutrophils located directly beneath the parakeratotic stratum corneum—within Munro’s microabscesses—appear to represent the primary source of IL-8 in the psoriatic tissue [41]. Given that IL-8 promotes neutrophil chemotaxis and that activated neutrophils are capable of producing IL-8, it is plausible that IL-8 functions in an autocrine manner to sustain neutrophil infiltration in psoriatic lesions, thereby perpetuating local inflammation [41].

Considering the anti-inflammatory properties of IL-11, a phase I open-label clinical trial investigated the efficacy of daily subcutaneous administration of recombinant human interleukin-11 (rhIL-11) in 12 patients diagnosed with psoriasis vulgaris [42]. After eight weeks of treatment, 11 out of 12 patients showed a 20–80% improvement in the Psoriasis Area and Severity Index (PASI), which was accompanied by a decreased expression of pro-inflammatory cytokines, including IFN-γ, IL-8, IL-12, TNF-α, and IL-1β. Large-scale clinical trials are warranted to validate the therapeutic potential of rhIL-11 as an anti-inflammatory agent in patients with psoriasis [34].

Ustekinumab exerts its therapeutic effects by targeting the p40 subunit, which is shared by both interleukin-12 (IL-12) and interleukin-23 (IL-23), two cytokines involved in inflammatory processes and in the activation of natural killer (NK) cells and CD4+ T lymphocytes in psoriasis. Moreover, both IL-23 and its receptor (IL-23R) are overexpressed in the psoriatic human tissue [43]. The distinct roles of IL-12 and IL-23 were explored in a mouse model of imiquimod-induced psoriasis [44]. As expected, mice deficient in the p40 subunit demonstrated a significant reduction in psoriatic plaque formation. However, genetically modified mice lacking the IL-12-specific p35 subunit or the IL-12 receptor—while maintaining intact IL-23 signaling—developed more severe inflammation and psoriatic lesions compared to wild-type controls. This exacerbated phenotype was associated with the increased infiltration of IL-17A-producing Vγ4+ γδT cells. Notably, the administration of IL-12 ameliorated this phenotype, suggesting a protective role of IL-12 in limiting skin inflammation in this murine model of psoriasis. In human keratinocytes, IL-12 exposure also reversed the psoriatic transcriptomic signature. Nevertheless, the exact role of IL-12 in patients with psoriasis remains uncertain, as no monoclonal antibody selectively targeting IL-12 has been evaluated in clinical settings. These findings raise the possibility that the simultaneous inhibition of both IL-12 and IL-23 may be counterproductive. In line with this, guselkumab and risankizumab—monoclonal antibodies targeting the p19 subunit specific to IL-23—have demonstrated superior clinical efficacy in patients with moderate-to-severe plaque psoriasis compared to ustekinumab [45,46].

Although the prevailing consensus is that Th17 cells are the primary source of IL-17 in psoriatic lesions, other cell types, such as mast cells and γδ T cells, also play significant roles in the pathogenesis of the disease [47,48]. IL-17 production by Th17 cells is stimulated by IL-23, which also promotes their survival [49]. IL-17, in turn, induces keratinocytes to produce antimicrobial peptides, along with various pro-inflammatory mediators, including cytokines (IL-1β, TNF-α, IL-6) and chemokines (CXCL1/3/5). The contribution of the IL-17/IL-23 axis to psoriasis pathogenesis has been explored in IL-17-deficient mice [50]. In wild-type mice, IL-23 exposure was sufficient to induce epidermal hyperplasia, whereas this effect was absent in IL-17-deficient mice. Furthermore, pre-treatment with anti-IL-17A antibodies prevented IL-23-induced epidermal hyperplasia. These findings suggest that IL-17A plays a critical role in IL-23-mediated psoriatic pathology. However, it remains unclear whether IL-17 upregulation alone is sufficient to initiate psoriatic disease, and whether this process can be effectively counteracted by IL-23-targeting antibodies or in an IL-23-knockout (IL-23^−/−^) context [34].

The IL-10 cytokine family includes IL-10, IL-28, IL-29, and members of the IL-20 subfamily—namely IL-19, IL-20, IL-22, IL-24, and IL-26 [51]. These cytokines play a significant role in the immunopathogenesis of psoriasis. Notably, IL-19 and IL-20 are overexpressed in the basal and suprabasal layers of psoriatic keratinocytes, although their circulating levels in the serum of psoriasis patients are reduced when compared to healthy controls [52,53]. Given that IL-19 is a key cytokine upregulated in response to IL-1β, its increased expression in psoriatic lesions may be a downstream effect of elevated IL-1β levels, as previously discussed [54]. Both IL-19 and IL-20 have been shown to induce the expression of keratinocyte growth factor (KGF) in CD8+ T lymphocytes. In a study evaluating matched synovial tissue and psoriatic skin samples from individuals with psoriatic arthritis (PsA) and plaque psoriasis, IL-20 expression was found to correlate positively with disease activity, as measured by the PASI, both at the baseline and after therapeutic intervention with alefacept [55].

IL-21 expression is significantly increased in psoriatic skin lesions in humans, and the intradermal administration of this cytokine in mice results in keratinocyte hyperplasia. Remarkably, treatment with anti-IL-21 antibodies was effective in alleviating inflammation and epidermal hyperplasia in a mouse model with human psoriatic skin xenografts. These results indicate that targeting IL-21 may represent a promising therapeutic strategy for the future management of psoriasis [34].

IL-22 expression is significantly increased in both psoriatic skin lesions and the serum of individuals with psoriasis when compared to healthy controls [56]. This interleukin is implicated in the regulation of antimicrobial defense proteins, such as psoriasin, calgranulin A, and calgranulin B, as well as in the modulation of keratinocyte migration through matrix metalloproteinases (MMP1 and MMP3). Additionally, the elevated plasma levels of IL-22 correlate positively with disease severity, as assessed by the PASI score, suggesting its involvement in the pathogenesis of psoriasis [57]. However, despite these findings, the monoclonal antibody targeting IL-22, fezakinumab, did not demonstrate efficacy in a phase 1 clinical trial [58,59].

IL-24 primarily targets the skin, where its receptors, IL-22R1/IL-20R2 or IL20R1/IL-20R2, are abundantly expressed. Furthermore, IL-24 expression is increased in psoriatic lesions and is produced by both peripheral blood mononuclear cells (PBMCs) and keratinocytes [54].

IL-25 (IL-17E) is significantly upregulated in human psoriatic lesions and is capable of inducing psoriasis-like pathological features in mice, such as acanthosis, parakeratosis, and immune cell infiltration. Importantly, IL-25 is stimulated by IL-17 and exerts positive autoregulatory effects in keratinocytes via the IL-17RB receptors, resulting in the increased production of pro-inflammatory cytokines and chemokines. This self-regulatory loop of IL-25 may present a promising therapeutic target for psoriasis, with potentially fewer adverse effects, particularly since its inhibition does not impair IL-17-mediated immune responses against infections [34].

IL-26 is markedly upregulated in psoriatic tissue and contributes to the processes of neovascularization and immune cell infiltration [60]. This cytokine has the capacity to induce human keratinocytes to produce the elevated levels of fibroblast growth factors, which are key mediators of angiogenesis. Notably, these growth factors are frequently elevated in the plasma of individuals with psoriasis and exhibit a positive correlation with disease severity [61].

The elevated serum concentrations of IL-27, IL-30, and IL-33 have also been observed in psoriasis patients and are significantly associated with disease severity, as determined by the PASI [62,63]. IL-29, a cytokine predominantly secreted by Th17 cells, is highly expressed in psoriatic skin lesions [64]. The in vitro stimulation of human keratinocytes with IL-27 or IL-29 results in the upregulation of chemokines such as CXCL9 and CXCL10, both of which are known to be overexpressed in psoriatic lesions [57].

Additionally, IL-32, IL-33, and IL-36α levels are increased in both the plasma and lesional skin of patients with psoriasis. However, their specific contributions to the immunopathogenesis of the disease remain to be fully elucidated [65,66].

Interleukin-37 (IL-37) and Interleukin-38 (IL-38) operate via distinct mechanisms in the pathogenesis of psoriasis, diverging from the pathways associated with previously characterized interleukins. IL-37 acts as a negative regulator of innate immune responses and exerts significant anti-inflammatory effects by suppressing the expression of key pro-inflammatory mediators, including CXCL8, IL-6, and S100A7, in human keratinocytes [67]. Furthermore, the exogenous administration of IL-37 has been shown to attenuate psoriatic manifestations in the K14-VEGF-Tg humanized mouse model of psoriasis [67]. Additional studies are necessary to delineate the full spectrum of IL-37’s immunomodulatory functions and to evaluate its potential as a therapeutic agent in human psoriasis [5].

IL-38, a receptor antagonist within the IL-36 cytokine family, exhibits reduced expression in both the serum and lesional skin of patients with psoriasis [67]. Interestingly, therapeutic intervention with secukinumab, an anti-IL-17A monoclonal antibody, results in the upregulation of IL-38 expression, a response that correlates positively with clinical improvement [67]. These findings underscore the relevance of IL-38 in psoriasis pathophysiology and support further investigation into IL-36 pathway inhibitors and recombinant IL-38 as potential therapeutic strategies.

### 3.5. Correlation Between Disease Severity and Serum Interleukin Levels

Currently, there is no established objective laboratory parameter for accurately assessing the severity of psoriasis. Th1-type cytokines have been found at elevated levels in both the lesional skin and the peripheral blood of patients with psoriasis. Serum, being easily accessible and requiring only small sample volumes for analysis, represents a practical medium for biomarker evaluation. Consequently, the measurement of serum cytokine levels may offer a more precise approach to monitoring disease progression and predicting clinical severity [68].

Recent advances in the understanding of psoriasis have highlighted the crucial role of both local and systemic cytokine regulation in the pathogenesis of the disease. The PASI remains the most widely used clinical scoring system for assessing disease severity. However, a simple laboratory test based on a blood sample represents a potentially valuable, patient- and observer-independent biomarker of disease activity. In this context, we reviewed several studies that investigated the association between the serum levels of pro-inflammatory cytokines measured in vivo and their correlation with psoriasis severity. Cytokine levels were determined using the enzyme-linked immunosorbent assay (ELISA) method. The mean serum concentrations of all analyzed cytokines were significantly elevated in patients compared to healthy controls. The following section presents significant correlations between the serum levels of interleukins involved in psoriasis and the clinical severity of the disease [5].

Psoriasis is recognized as a T cell-mediated disorder, characterized by the involvement of a wide range of cytokines, which has led to the development of novel immunomodulatory therapies. Numerous studies have demonstrated that the serum levels of TNF-α, IFN-γ, IL-6, IL-8, IL-12, IL-18, and IL-30 are significantly elevated in patients with active psoriasis compared to healthy individuals. Furthermore, the elevated serum levels of IFN-γ, IL-12, and IL-18 have shown significant correlations with clinical severity and disease activity. These findings suggest that the measurement of these serum cytokines may serve as objective parameters for assessing the severity of psoriasis [68]. Notably, consistent with previous studies investigating cytokines in psoriasis [68,69], this study found no significant association between serum IL-30 levels and either patient age or sex. Similarly, neither age at diagnosis nor disease duration showed a statistically significant relationship with the mean rank of serum IL-30 levels. These findings are in agreement with the existing literature regarding the association of related cytokines with psoriasis [69,70,71].

In the study conducted by Ozer Arican et al. [68], the findings corroborated previously published data, indicating that Th1-associated cytokines (TNF-α, IFN-γ, and IL-12), along with several pro-inflammatory cytokines (including IL-6, IL-8, and IL-18), exhibit altered serum levels in patients with psoriasis. Furthermore, the authors reported a significant correlation between disease severity and the serum concentrations of IFN-γ, IL-12, IL-17, and IL-18. Their results also demonstrated that the serum levels of these cytokines were not significantly associated with patients’ age or gender.

Previous research has consistently shown the elevated serum concentrations of IFN-γ [72,73,74], TNF-α [75,76], IL-8 [72,77,78,79,80], and IL-18 [81] in psoriatic patients compared to healthy controls. Although the precise role of TNF-α in the pathomechanism of psoriasis remains to be fully elucidated, the high therapeutic efficacy of TNF-α inhibitors strongly supports the pivotal role of this cytokine—alongside IFN-γ—in the disease’s pathogenesis. Both IFN-γ and TNF-α are known to induce the expression of IL-6, IL-8, IL-12, and IL-18, thereby constituting a key regulatory axis within the cytokine network implicated in psoriasis [6]. Notably, the intradermal administration of IFN-γ into non-lesional psoriatic skin has been shown to provoke the development of lesions at the injection site [82].

Interleukin-6 (IL-6) is a pro-inflammatory cytokine that promotes the proliferation of dermal and epidermal cells [83]. IL-6 is known to facilitate T cell activation and stimulate keratinocyte proliferation [84], while also playing a crucial role in the acute-phase response during early inflammation [85]. Multiple studies have demonstrated that serum IL-6 levels serve as a critical biomarker for assessing both treatment response and the degree of systemic inflammation [39,86]. Therefore, the findings reported by Bincy Verghese et al. regarding elevated IL-6 serum levels in patients with psoriasis are consistent with the critical role of this cytokine in the pathogenesis of the disease. The difference in IL-6 serum levels between patients and healthy controls was highly significant (*p* < 0.001). This finding is consistent with previous studies conducted by Abanmi et al. [77] and Koliadenko et al. [87], both of which reported elevated IL-6 levels in their patient cohorts. However, contradictory results were reported by Jacob et al. [72], who observed no significant difference in serum IL-6 concentrations between psoriatic patients and controls [88]. In patients with psoriasis, elevated IL-6 concentrations have been correlated with the severity of cutaneous and joint involvement, as IL-6 plays a key role in sustaining inflammation and promoting synovial hyperplasia [89].

The elevated levels of IL-8 have been identified in the psoriatic lesional skin, and numerous studies suggest that IL-8 contributes to psoriasis pathogenesis [90]. The available evidence indicates that IL-8 functions as a potent chemoattractant for neutrophils and T lymphocytes, while also promoting keratinocyte proliferation [82]. Teranishi et al. investigated the spontaneous production of IL-8 by peripheral blood monocytes in patients with psoriasis, in comparison to healthy controls, and also reported the elevated serum levels of IL-8 in psoriatic patients [91]. In contrast, Sticherling et al. [90], utilizing both in-house assays and three commercial ELISA kits, found no correlation between serum IL-8 concentrations and disease severity at any stage of psoriasis. Due to the presence of down-regulatory mechanisms, it can be hypothesized that, in cases of prolonged disease relapse, correlations between clinical parameters and plasma IL-8 concentrations may no longer be detectable [92].

In their study, Sharon E. Jacob et al. provided findings that challenge previous reports by demonstrating a significant elevation of IL-8 levels in patients with psoriasis. The increased serum concentration of IL-8 was found to positively correlate with the degree of erythema. IL-8 is a well-characterized chemotactic cytokine for neutrophils and indirectly promotes neutrophil degranulation. Given the presence of neutrophils within the epidermis of psoriatic lesions, it is plausible that IL-8 facilitates their recruitment and, consequently, contributes to the development of erythema. Although the serum kinetics of IL-8 in psoriasis remain incompletely understood, these findings suggest that IL-8 may serve as a valuable biomarker of the inflammatory process and a key mediator in the cellular activation underlying psoriatic pathology [72].

Sharon E. Jacob et al. reported a significant reduction in serum IL-10 levels in patients with psoriasis, with this marked suppression representing a consistent finding. IL-10 is a well-established anti-inflammatory cytokine that inhibits macrophage-derived cytokine and chemokine production, while promoting the release of soluble cytokine receptors. Furthermore, IL-10 is known to modulate antigen presentation by dendritic cells and suppress co-stimulatory signaling through its direct effects on T cells.

In a related study, McInnes et al. demonstrated that a 28-day course of subcutaneous administration of recombinant human IL-10 resulted in a clinically significant improvement of cutaneous lesions—but not joint symptoms—in 29 patients with psoriatic arthritis. Additionally, treatment with recombinant IL-10 led to selective functional suppression of pro-inflammatory activity in circulating monocytes [93].

Low IL-10 levels have been observed in psoriatic skin lesions, and elevated IL-10 mRNA levels have been reported in peripheral blood mononuclear cells following antipsoriatic treatment. These findings align with the observed decrease in serum IL-10 levels in the patient cohort of Jacob et al.’s study. Interestingly, patients with psoriatic arthritis have been shown to exhibit elevated IL-10 serum levels, which may represent a compensatory mechanism aimed at modulating systemic inflammation in individuals with both cutaneous and articular involvement [72].

IL-12 is critical for the development of Th1-mediated immune responses and may also play a role in initiating new psoriatic lesions in situ [94].

In their study, Sharon E. Jacob et al. concluded that IL-12 levels did not show a significant increase in patients with psoriasis compared to healthy controls. On the contrary, IL-12 was significantly decreased in psoriatic patients. A normal level of IL-2 was expected, as this cytokine is known to stimulate the proliferation of activated T and B lymphocytes. However, the finding that IL-12 was not elevated, but rather reduced, was unexpected given its critical role in the differentiation of Th1 cells. IL-12 promotes the proliferation of Th1 lymphocytes and their production of IFN-γ. The observation that IFN-γ levels were elevated in psoriatic patients despite reduced IL-12 suggests that IFN-γ production in psoriasis may be driven by an alternative regulatory pathway [72].

The pleiotropic impact of IL-16 on immune system cells and its association with CD4+ lymphocytes suggest that this cytokine may be involved in the pathogenesis of psoriasis. There are only a few studies in the literature addressing IL-16 in this disease. The aim of the study by Dorota Purzycka-Bohdan et al. was to investigate whether the serum levels of IL-16 and the cutaneous mRNA expression of IL-16 (messenger RNA) correlate with the clinical severity of psoriasis and the cutaneous mRNA expression of CD4. Additionally, the genotype and allele frequencies for the -295 T/C promoter gene polymorphism of IL-16 were analyzed. The serum levels of IL-16 were significantly elevated in patients with psoriasis compared to unaffected individuals. Additionally, IL-16 serum levels exhibited a positive correlation with the PASI and Body Surface Area (BSA), and were notably higher in patients with moderate-to-severe psoriasis. No statistically significant differences were observed in IL-16 serum levels between patients with mild psoriasis and control subjects, nor between early-onset and late-onset psoriasis. Furthermore, no significant correlation was found between IL-16 serum levels and quality of life, as assessed by the Dermatology Life Quality Index (DLQI) [95].

The literature presents conflicting evidence concerning serum IL-17 levels in patients with psoriasis. The majority of studies have found no significant differences in IL-17 serum concentrations between psoriatic patients and healthy control groups [68,96,97]. Conversely, Takahashi et al. [98] and El-Moaty Zaher et al. [99] recently reported significantly elevated IL-17 levels in the sera of psoriatic patients compared to controls. In agreement with El-Moaty Zaher et al. [99], but in contrast to other findings [68,96,97], the study conducted by Aikaterini Kyriakou et al. suggested that elevated IL-17 levels do not correlate with the PASI. Nevertheless, it is noteworthy that all studies—except that of Takahashi et al. [98]—reported a significant correlation between IL-17 and the PASI, despite the absence of significant differences in IL-17 serum levels between patient and control groups.

There is clearly a lack of consensus concerning the relationship between IL-17 serum levels and disease severity as assessed by the PASI. This inconsistency is most likely attributable to heterogeneity in inclusion criteria and study populations. IL-17 serum levels may be elevated due to cutaneous, articular, or nail involvement in psoriatic disease, whereas the PASI reflects only the severity of skin manifestations. Within this context, the correlation between the PASI and cytokine serum levels may also be misleading. Therefore, it is generally accepted that the most valid results are obtained when analyses are restricted to patients presenting exclusively with cutaneous lesions [69].

In the same study conducted by Ozer Arican et al., the authors observed that serum IL-17 levels were slightly elevated in patients with psoriasis compared to healthy controls; however, this difference did not reach statistical significance. IL-17, a cytokine secreted by activated CD4+ T lymphocytes, acts synergistically with IFN-γ to enhance the production of pro-inflammatory cytokines such as IL-6 and IL-8 by human keratinocytes, thereby promoting the recruitment of T cells to the skin. IL-17 itself is also classified as a pro-inflammatory cytokine. Its effect is further potentiated in the presence of IFN-γ, with costimulatory signaling leading to the increased secretion of IL-6 and IL-8 [100]. Despite this, in vivo data regarding the biological role of IL-17 remain limited [101]. Although IL-17 serum levels appeared within the normal range in the psoriatic cohort studied, a significant correlation was still observed between IL-17 levels and the PASI score. Additional studies are warranted to confirm these findings and to further elucidate the in vivo role of IL-17 in the pathogenesis of psoriasis.

Interleukin-18 is a cytokine potentially involved in the pathogenesis of psoriasis. In their study, Aldona Pietrzak et al. investigated peripheral blood lymphocyte subpopulations and the expression of their activation markers, correlating these findings with plasma IL-18 levels and the clinical severity of the disease in patients with psoriasis [102].

They found that the levels of IL-18 were significantly elevated in patients with psoriasis compared to healthy subjects. A significant correlation was observed between IL-18 levels and the psoriatic lesion surface area (*p* < 0.04) and the PASI score (*p* < 0.03) [102].

IL-18 has been shown to exhibit a wide range of biological activities. As a pleiotropic cytokine, it may play an immunoregulatory role in the human body’s defense system, particularly in inflammatory, infectious, and autoimmune diseases [103]. IL-18 promotes angiogenesis and tumor suppression and has been found to be elevated in nearly all inflammatory diseases—from psoriasis and atopic eczema to pancreatitis and infections [104,105,106,107,108,109,110,111].

Naik et al. [112] observed that, among skin cells not derived from the bone marrow, human keratinocytes constitutively express IL-18 mRNA, whereas dermal endothelial cells, dermal fibroblasts, and melanocytes do not. The levels of IL-18 mRNA and intracellular protein are not influenced by pro-inflammatory stimuli, neither in human keratinocytes nor dermal cells. Immunohistochemical analysis revealed IL-18 protein in the basal keratinocytes of normal skin, with a marked increase in expression in suprabasal keratinocytes in psoriasis patients [113,114].

Both Ohta et al. [115] and McKenzie et al. [113] reported an increase in IL-18 concentration and its receptor in both the lesional and non-lesional skin in psoriasis, suggesting its involvement in the psoriatic process. The elevated plasma levels of IL-18 observed in the study by Aldona Pietrzak et al. may indicate a systemic activation of this cytokine’s production [102].

IL-20 is synthesized by keratinocytes in response to stimulation by IL-22, TNF-α, and IL-17, but not by IFN-γ or IL-20 itself [116,117]. In addition, activated monocytes and dendritic cells are also capable of producing IL-20 [54,118]. This cytokine appears to play a critical role in the late effector phase of psoriasis pathogenesis, where it contributes to the inhibition of terminal keratinocyte differentiation, enhances antimicrobial defense mechanisms, and promotes the production of neutrophil-attracting chemokines within keratinocytes [117,119].

Although studies on serum IL-20 levels are limited, elevated concentrations have been documented both in the lesional skin and in the peripheral blood of patients with psoriasis [117]. Consistent with the findings of the study conducted by Anna Michalak-Stoma et al. [97], serum IL-20 levels were found to correlate with the PASI scores, indicating a potential link between IL-20 expression and disease severity. The significantly elevated levels of IL-20 were identified in patients with psoriasis compared to the control group (*p* < 0.001). A statistically significant positive correlation was observed between IL-20 concentrations and psoriasis severity, as assessed by the PASI score (*p* < 0.001).

In their study, Bartłomiej Wawrzycki et al. [120] reported the elevated serum levels of IL-22 in patients with psoriasis compared to healthy controls, consistent with previous findings [56,121]; Shimauchi et al. [122]; Wolk et al. [58]. Moreover, IL-22 levels were positively correlated with disease severity as measured by the PASI, supporting the notion that IL-22 expression is not merely a bystander phenomenon but may play an active role in the pathogenesis of psoriasis.

The initial evidence linking IL-22 to psoriasis pathogenesis emerged from genetic studies. A specific IL-22 variant, associated with enhanced epithelial barrier defense, was found to be preferentially enriched in a studied population and linked to earlier disease onset [123,124].

Interleukin-30 (IL-30) is a 28 kDa protein that belongs to the IL-6 cytokine family [125,126]. It is primarily produced by myeloid-derived immune cells, including macrophages, monocytes, microglia, and dendritic cells, in response to various microbial and immunological stimuli. Additionally, IL-30 is secreted by neutrophils, plasma cells, endothelial cells, and epithelial cells [127,128]. It participates in the activation of both classical and trans-signaling pathways, indicating its potential to exert diverse effects on multiple cell types [129,130].

IL-30 has been shown to play an independent and significant role in the development and progression of autoimmune diseases, such as psoriasis, by modulating T helper type 1 (Th1) and Th17 cell responses [131]. Moreover, previous research has suggested that the measurement of serum cytokine levels in patients with psoriasis enhances the understanding and prediction of disease pathology [132]. Consequently, the assessment of serum IL-30 concentrations may provide valuable insight into the disease course and prognosis in individuals with psoriasis. Another study investigated serum IL-30 levels in patients with psoriasis and evaluated their correlation with disease severity [71].

This study demonstrated that serum IL-30 levels were significantly elevated in patients with psoriasis compared to healthy controls. Furthermore, the findings indicated a positive correlation between serum IL-30 concentrations and psoriasis severity as assessed by the PASI score, although this correlation did not reach statistical significance. Despite known limitations of the PASI score, including its non-linear scale [133,134] and limited sensitivity [135], it remains the most widely utilized clinical tool for assessing psoriasis severity [135].

Several studies in the literature have investigated the circulating levels of various cytokines in the serum of patients with psoriasis and compared these findings with those observed in healthy controls [68,72,75,77,88,98,132,136,137]. The majority of these studies have reported the significantly elevated serum levels of tumor necrosis factor-alpha in psoriatic patients compared to healthy individuals [68,75,77,88,98,132,136,138,139]. However, findings by Tigalonova et al. [137] and Jacob et al. [72] contradict this trend, as they reported no significant differences in TNF-α serum levels between psoriatic patients and controls.

Furthermore, there is no consistent consensus in the literature regarding the correlation between serum TNF-α levels and disease severity, as measured by the PASI score. While several studies have failed to identify a significant association between TNF-α levels and the PASI scores [68,132,138], other investigations have reported a positive correlation [75,98].

The significance of serum cytokines in dermatology is experiencing a marked increase. Novel ELISA-based techniques offer high specificity and reliable standardization, although their application remains limited by elevated costs [140]. Nevertheless, cytokine assay outcomes may vary depending on the analytical methods employed, their sensitivity, potential interference from administered pharmacological agents, and the influence of concomitant comorbidities [141].

The skin, as the largest organ of the human body, serves as the site for more than 50 clinically distinguishable inflammatory dermatologic diseases. Consequently, the precise role of circulating cytokines remains to be fully elucidated, and further investigations are warranted, particularly regarding the pathogenesis of cutaneous conditions such as psoriasis. A central unresolved question is whether cytokine dysregulation represents a primary pathogenic trigger or a secondary phenomenon—the “chicken or the egg” dilemma. The current evidence suggests that these alterations are more likely a consequence rather than a direct cause of dermatologic disorders.

A comprehensive understanding of the in vivo immunologic cascade, as previously outlined, is essential for the advancement of targeted biologic therapies. It is yet to be definitively established whether the inhibition of specific cytokine activity, through monoclonal antibodies or selective cytokine inhibitors, will translate into novel therapeutic strategies [142,143]. Until additional inflammatory dermatoses are thoroughly investigated, the specificity and broader applicability of current findings remain uncertain. Thus, further research is essential to clarify the pathogenic role of serum pro-inflammatory cytokines and their correlation with clinical disease severity in psoriasis [68].

With a more comprehensive understanding of the interactions between cytokines, it may become possible to more accurately characterize the pathogenesis of the disease. In diagnostically challenging cases, as well as in patient follow-up, the simultaneous assessment of multiple cytokine parameters in peripheral blood may prove to be a valuable tool for clinical evaluation and disease monitoring.

#### Fluctuations in Cytokine Levels During Exacerbations and Remissions in Psoriasis

The relapsing–remitting nature of psoriasis poses a substantial challenge to the diagnostic reliability of salivary cytokine biomarkers, primarily due to the temporal variability in immune system activity. Psoriasis is characterized by the alternating phases of active inflammation (exacerbation) and clinical remission, during which cytokine expression levels fluctuate significantly [144].

Key pro-inflammatory cytokines, such as interleukin-17 (IL-17), interleukin-23 (IL-23), and tumor necrosis factor-alpha (TNF-α), are markedly elevated during disease flares but often decrease to near-baseline levels during remission. This dynamic expression pattern can adversely affect the sensitivity and specificity of salivary biomarkers when samples are collected without accounting for the disease phase. Consequently, biomarkers that demonstrate high discriminatory power during active disease may produce false-negative results during remission, thus limiting their overall diagnostic value [145].

Additionally, saliva as a diagnostic medium introduces inherent complexities due to its dynamic biochemical composition, which is influenced by factors such as circadian rhythm, oral health status, and psychological stress—all of which can confound cytokine quantification. When coupled with the episodic nature of psoriasis, these variables further underscore the necessity for standardized sample collection protocols and contextual interpretation of biomarker data [146].

To mitigate these limitations, longitudinal sampling across multiple disease states is recommended. The use of composite biomarker panels, rather than reliance on individual cytokines, in conjunction with clinical severity indices (e.g., PASI score) may enhance diagnostic robustness. Moreover, the application of machine learning models to analyze cytokine dynamics across disease phases offers promising avenues for improving predictive accuracy and individualized disease monitoring [147]. We have included “Serum cytokine alterations and their clinical significance in psoriasis” in Table 1 to summarize the key findings from Section 3.5 in the form of a table for each cytokine.

### 3.6. Salivary Interleukins—Potential Biomarkers in Psoriasis

A scoping review conducted by Larissa Conrado da Silva et al. [143] systematically synthesized the existing evidence regarding salivary biomarkers in patients with psoriasis. A total of 22 studies were included, encompassing cross-sectional, cohort, and randomized clinical trial designs, involving a combined sample of 987 individuals. The most frequently assessed salivary biomarkers included pro-inflammatory cytokines (e.g., IL-1β, IL-6, TNF-α), oxidative stress indicators (e.g., nitric oxide, nitrotyrosine), antioxidant enzymes, immunological markers (e.g., salivary alpha-amylase, secretory immunoglobulin A), and stress-related hormones such as cortisol. Several of these biomarkers demonstrated significant associations with both disease severity and therapeutic outcomes. Nonetheless, the interpretation of the findings is constrained by methodological heterogeneity and the absence of standardized protocols. The review highlights the promise of salivary diagnostics in the context of psoriasis and calls for future high-quality, adequately powered studies to validate and expand upon these preliminary findings.

### 3.7. Advantages of Saliva as an Analytical Medium (Non-Invasive, Repeatable, Cost-Effective)

Unlike blood tests, which require specific materials for biological sample collection and trained personnel, saliva collection is a painless and straightforward procedure, offering a viable alternative especially for children and the elderly, while also facilitating at-home testing [148]. The simplicity of saliva collection also confers significant logistical benefits [59,149,150].

The demand for rapid and minimally invasive diagnostic tests has surged significantly over the past decade, resulting in extensive research on saliva as a biological fluid for clinical diagnostics [151,152]. Saliva presents several advantages over blood and urine, two of the most commonly utilized diagnostic fluids in laboratory settings. Saliva collection is straightforward and non-invasive, requiring relatively simple instructions, and it features lower protein content, reduced complexity, and a more variable composition compared to serum [153,154]. Salivary DNA is routinely used in numerous clinical laboratories to assess genetic predispositions to various diseases. Saliva-based diagnostic tests have been effectively employed in the diagnosis of human immunodeficiency virus infection [155], monitoring of renal diseases [156], prevention of cardiometabolic risks [157], detection and quantification of viral nucleic acids [158], forensic medicine investigations [159], dental research [160,161], and drug abuse monitoring [159].

Early disease detection is essential for effective clinical management, as it increases survival rates and minimizes the risk of complications [162]. Monitoring during the initial phase allows for timely intervention, potentially preventing disease progression and improving overall patient outcomes. A key objective in healthcare research is the ability to assess physiological conditions, track disease progression, and monitor post-treatment therapeutic outcomes through non-invasive methods. Saliva, a complex oral fluid that can be collected using non-invasive techniques, holds considerable potential for evaluating both general health and specific diseases. It contains a wide range of proteins and peptides, each playing significant biological roles [154]. With recent advancements in technologies such as bioinformatics, metabolomics, genomics, and proteomics, saliva has become an increasingly valuable clinical tool due to its capacity to reflect both oral health status and systemic health conditions [163]. However, for saliva-based diagnostics to be effective, two critical prerequisites must be met, i.e., A. the identification of specific biomarkers for various diseases within the complex composition of saliva, and B. the rigorous evaluation of their sensitivity and specificity through ongoing research and methodological advancements [164].

Saliva can be employed to detect not only viruses, bacteria, and other biomarkers, but also a variety of molecular and genetic markers for the early detection, treatment, and monitoring of cancer and other diseases. Saliva-based diagnostic tests have demonstrated high sensitivity (≥95%) and specificity, reflecting the ability of the test to accurately identify true-positive and true-negative cases [165].

Saliva holds significant potential as a convenient, non-invasive diagnostic fluid, offering notable advantages over other biological samples such as blood, smears, and tissue biopsies in terms of cost, ease of collection, multiple sampling opportunities, and reduced risk of transmitting infectious agents to healthcare personnel. It is particularly valuable for monitoring systemic health, disease progression, and prognosis. Salivary biomolecules can serve as reliable diagnostic tools for detecting various malignancies, genetic disorders, and hormonal abnormalities. Additionally, salivary diagnostics can aid in identifying oral infections, the oral microbiome, metabolites, genetic mutations, epigenetic alterations, cytokines, immunoglobulins, hormones, mucins, growth factors, inhibitors, enzymes, non-coding RNAs, and salivary exosomes and exosomal miRNAs [159,165,166]. Therefore, saliva is considered an invaluable therapeutic biofluid, as it contains a range of biologically significant molecules and macromolecules that can easily function as biomarkers for disease diagnosis, prognosis, and therapeutic targets, facilitating effective treatment and monitoring of both oral and systemic diseases. Salivary testing is among the simplest of all available diagnostic methods, as it does not cause discomfort, pain, toxicity, or inconvenience, and is a cost-effective, widely used procedure for common screening [165].

#### Standardization of Saliva Collection

Although salivary inflammatory cytokines have been increasingly incorporated into research across biobehavioral, psychological, clinical, and public health domains, guidance regarding standardized procedures for the collection, processing, and storage of biospecimens remains limited, potentially compromising the accuracy and validity of the resulting data. Moreover, associations between salivary cytokines and indicators of oral health status are rarely examined and often overlooked in studies conducted outside the field of dentistry, which may introduce significant confounding factors in the interpretation of findings [167].

The commonly reported limitations of saliva collection include diurnal variability and the inconsistent timing of sampling, the potential for blood contamination, and the confounding influence of suboptimal oral health [168].

Pre-collection fasting is recommended to minimize the risk of contamination with food residues, enzymatic activity alterations, and transient fluctuations in cytokine concentrations. Most standardized protocols advise a fasting interval ranging from 30 min to 2 h prior to saliva collection, during which participants should refrain from consuming food or beverages (with the exception of water), smoking, and using oral hygiene products [169].

Salivary cytokine concentrations exhibit circadian variation, with the peak levels of pro-inflammatory cytokines frequently observed in the early morning hours. To minimize temporal variability and enhance data consistency, it is recommended that saliva samples be collected at a standardized time of day, preferably between 8:00 and 10:00 a.m. [170].

Unstimulated whole saliva is generally preferred for cytokine analysis because it minimizes dilution effects and reduces variability. However, in populations characterized by diminished salivary flow—such as elderly individuals or psoriatic patients with comorbidities—mild mechanical or gustatory stimulation may be necessary to obtain adequate sample volumes. When stimulation is employed, it should be carefully standardized, for instance, by using chewing gum. The use of citric acid or variable types of chewing gum is discouraged due to their inconsistent stimulatory effects and potential immunomodulatory properties that may confound cytokine measurements [171].

Validated commercial assays like ELISA and multiplex bead-based platforms are widely employed for their standardized protocols, high reproducibility, and regulatory validation. Their rigorous performance checks for specificity and sensitivity, combined with simplified procedures, help minimize technical variability—critical for saliva samples that often have low analyte levels and are prone to enzymatic degradation [172,173].

However, cytokine stability in saliva can be highly variable due to factors such as pH, protease activity, and circadian influences [174]. For this reason, customized protocols may be necessary to optimize pre-analytical variables, including the use of protease inhibitors, specific collection devices, or immediate sample freezing, which are not always addressed by commercial kits.

In the context of psoriasis, where cytokines such as IL-17 and IL-23 may be present at low concentrations or exhibit instability in saliva, protocols customized to account for the specific half-life and stability of these cytokines can improve assay sensitivity and enhance the reliability of the data obtained [175]. Therefore, while commercial kits are advantageous for standardization, customized approaches may be warranted to ensure the integrity of labile cytokines in salivary matrices.

An integrative strategy should be adopted, combining the use of validated commercial kits with customized pre-analytical protocols specifically designed to address cytokine stability in saliva. This hybrid approach optimizes both reproducibility and analytical accuracy in salivary cytokine profiling for psoriasis research.

### 3.8. Rationale for Using Saliva as an Alternative Biological Fluid

Human saliva is a biologically complex fluid secreted by the salivary glands and gingival tissues, containing a wide range of components such as proteins—including cytokines—alongside various organic and inorganic molecules essential for preserving oral homeostasis [154]

Beyond its physiological role, saliva has emerged as a promising diagnostic fluid, offering significant advantages over traditional biological samples, primarily due to its non-invasive, cost-effective, and easily accessible collection method. Recent evidence indicates that salivary cytokines serve as valuable biomarkers, providing insights not only into local oral health conditions but also reflecting the presence and activity of systemic diseases [176].

Saliva has emerged as a valuable and non-invasive alternative biological fluid for biomarker analysis in various systemic inflammatory and autoimmune diseases, including psoriasis. The use of saliva offers several advantages over conventional blood sampling, particularly in the context of monitoring cytokines such as interleukins, which play a pivotal role in the immunopathogenesis of psoriasis [162].

Firstly, saliva collection is simple, safe, non-invasive, and cost-effective, making it suitable for repeated sampling, especially in vulnerable populations or in long-term disease monitoring. This minimizes patient discomfort and enhances compliance compared to invasive procedures such as venipuncture [150].

Secondly, saliva reflects systemic immune and inflammatory status due to the presence of locally produced and transudated biomarkers from blood circulation through the salivary glands. Several studies have demonstrated that cytokines, including interleukins like IL-17, IL-23, and IL-6, can be reliably detected in saliva, correlating with their serum levels in inflammatory conditions.

Recent studies have employed advanced technologies that allow for the simultaneous quantification of multiple cytokines within a minimal volume of saliva.

### 3.9. Existing Studies on Salivary Interleukins in Psoriasis

To date, numerous biomarkers have been proposed for psoriasis; however, none have been validated or widely accepted as definitive disease markers.

An ideal biomarker is a biological indicator that is sensitive, specific, reproducible, and capable of accurately reflecting a physiological or pathological condition and/or a therapeutic response [177]. Moreover, the assay used to evaluate such biomarkers should be validated, standardized, and easily implementable in clinical settings [177].

In recent years, biomarker research in psoriasis has predominantly focused on blood and skin samples, as well as genetic and transcriptomic analyses, often yielding inconsistent and inconclusive results [178]. Consequently, saliva—with its dual secretory pathways—has emerged as a promising, non-invasive, and accessible alternative biological fluid for biomarker discovery [179,180,181]. Initial evidence, primarily originating from research in rheumatic diseases, highlights a significant association between salivary composition, oral inflammation, and systemic health. Additionally, the oral microbiome has been shown to influence both cutaneous and gastrointestinal systems, thereby contributing to systemic inflammatory processes [182,183].

Krasteva et al. found no statistically significant differences in salivary IgA levels—measured using radial immunodiffusion—between patients with psoriasis and healthy controls. However, they observed a trend toward lower IgA levels in patients with a PASI score greater than 10, compared to those with a PASI score below 10, suggesting that individuals with more severe disease may be at an increased risk for microbial infections that could trigger or exacerbate psoriasis [184].

In the same study, the authors reported a statistically significant increase in salivary C-reactive protein (CRP) levels, consistent with the inflammatory nature of psoriasis. As previously established, elevated CRP has prognostic relevance in predicting disease progression [42]. Furthermore, increased salivary haptoglobin levels were also documented, indicating a potential local defense response in the context of psoriasis [185].

While salivary biomarkers have been identified across a range of systemic diseases, the current evidence supporting their association with psoriasis remains limited [186,187]. Within this framework, Ganzetti et al. assessed the salivary expression levels of several cytokines and chemokines—including interleukin IL-1β, IL-6, transforming growth factor β1, IL-8, TNF-α, IFN-γ, IL-17A, IL-4, IL-10, monocyte chemoattractant protein MCP-1, macrophage inflammatory protein MIP-1α, and MIP-1β—utilizing multianalyte ELISA arrays in patients with psoriasis [188].

Their analysis revealed a statistically significant upregulation of TNF-α, TGF-β1, MCP-1, and IL-1β in the psoriatic saliva compared to that of healthy individuals. Additionally, IL-1β, TGF-β1, and MCP-1 levels were positively correlated with the clinical severity of oral involvement [188]. It has been proposed that the elevated expression of IL-1β in psoriatic patients may underlie the increased incidence of tooth loss, alveolar bone resorption, and periodontitis reported in this population [189].

Periodontitis, a progressive inflammatory condition of the periodontal supporting tissues, is primarily driven by bacterial biofilms [189]. The aforementioned findings suggest a shared inflammatory mechanism between psoriasis and periodontal disease, with IL-1β playing a pivotal role in tissue degradation, potentially via the upregulation of matrix metalloproteinases [190].

The observation of increased salivary IL-1β levels in individuals with psoriasis was corroborated by Mastrolonardo et al., who reported elevated basal IL-1β concentrations, indicating enhanced cytokine activity in these patients [191]. Such alterations in cytokine dynamics are thought to contribute to the chronic inflammatory milieu characteristic of psoriatic skin [183].

Moreover, the involvement of oral mucosa in psoriasis may be further substantiated by the elevated levels of TGF-β1 and MCP-1, both in the saliva [188] and in the serum of affected individuals [192,193].

In a separate investigation, Ganzetti and colleagues validated the utility of saliva as a non-invasive biomatrix for monitoring inflammatory responses in psoriasis [194]. Baseline assessments demonstrated significantly elevated salivary IL-1β levels in psoriatic patients, as measured by the enzyme-linked immunosorbent assay (ELISA), relative to healthy controls. Following 12 weeks of anti-TNF-α therapy, IL-1β concentrations declined significantly from the baseline, yet remained elevated compared to control values. These findings underscore the potential of salivary IL-1β as a biomarker for disease monitoring, although further studies with larger cohorts are required to confirm its clinical applicability.

### 3.10. Role of Interleukins in Monitoring Treatment Response

The selection of the most effective therapeutic approach in psoriasis remains a significant clinical challenge, frequently requiring multiple treatment attempts. Typically, this involves an initial trial of topical therapies, followed by the addition of systemic agents, and ultimately the transition to biologic therapies. Clinically, it is often observed that optimal outcomes are achieved primarily through the use of biologic agents. Most existing studies in the literature focus on assessing and comparing cytokine levels between psoriasis patients and healthy controls. However, few investigations have addressed the longitudinal effects of treatment on cytokine levels within the same cohort of psoriasis patients [195].

The study conducted by Choe et al. demonstrated a statistically significant correlation between the PASI score and IL-17A levels across the patient population, although no such association was found with IL-23 [196]. This observation was restricted to patients with stable disease and did not extend to those with erythrodermic forms. Conversely, the study by Bilgiç et al. reported no correlation between serum cytokine levels and either disease severity or duration [197]. Nonetheless, their findings indicated the elevated serum levels of pro-inflammatory cytokines IL-6, IL-23, and TNF-α in psoriatic patients compared to healthy individuals.

Cataldi et al. found no significant influence of age or sex on cytokine levels among psoriasis patients [198]. Using relative quantification techniques, Kutwin and colleagues reported the increased gene expression of IL-23A in patients with PASI scores greater than 10 compared to those with lower scores [199]. However, this was not reflected in the DLQI score. Furthermore, no statistically significant correlations were identified between the gene expression of IL-17A, its receptor, or the IL-23 receptor, and either the PASI or DLQI scores. In their cohort, no significant associations were observed between cytokine levels and the PASI scores, and an unexpected inverse correlation was noted between IL-23 levels and the DLQI scores. This anomaly may be attributable to a statistical error resulting from the limited sample size.

Following three months of treatment with biologic agents, IL-23 levels exhibited a statistically significant decrease, while reductions in TNF-α and IL-17F approached statistical significance. According to Philipp et al., the serum concentrations of IL-17A, IL-17F, and IL-22 were significantly reduced at both 12 and 52 weeks after initiating ustekinumab therapy [200]. Similarly, significantly lower levels of IL-6 and IL-22 were observed after 36 months of biologic therapy [201]. These findings suggest that more pronounced reductions in cytokine levels may become evident over extended treatment periods (12–24 months).

In another study by Bramsen Andersen et al., statistically significant reductions in IL-23, IL-17, and TNF-α were reported at 3 months post-initiation of biologic therapy [202]. Additionally, a separate study evaluating the cardiovascular benefits of ustekinumab revealed a trend toward a significant reduction in IL-17A and IL-23 levels at 12 weeks (*p* < 0.1), with more substantial decreases observed by week 52 [203].

In contrast, anti-TNF-α immunomodulatory therapies did not significantly affect pro-inflammatory cytokine levels in the study by Cataldi et al. [198]. Thus, despite promising evidence, several uncertainties persist regarding the specific effects of biologic therapies on cytokine profiles in psoriasis.

The study conducted by Florian L. et al. [204] clearly demonstrated a statistically significant or near-significant reduction in IL-23, TNF-α, and IL-17F, along with a notable decrease in the ESR, CRP, PASI, and DLQI scores, supporting the clinical and immunological efficacy of biologic treatments in moderate-to-severe psoriasis.

The use of salivary ILs could enhance early detection, monitor therapeutic response, and predict relapse. However, the standardization of collection methods, circadian variations, and individual differences in salivary composition pose challenges [195]. Longitudinal studies and larger cohorts are needed to validate specific thresholds and biomarker panels.

Several interleukins including IL-17A, IL-22, IL-23, IL-36γ, IL-6, and IL-1β have emerged as the potential biomarkers of subclinical inflammation and treatment resistance in psoriasis. The persistent elevation of these cytokines, even during clinical remission, suggests ongoing immune activation and a higher risk of relapse or therapeutic failure. Their monitoring, particularly through non-invasive methods, may enhance the early detection of immune dysregulation and support personalized treatment strategies [120,144,205,206,207].

### 3.11. Future Research Directions—Standardization of Saliva Collection and Analysis Methods

Salivary diagnostics offer considerable advantages owing to their non-invasive nature and the ease with which samples can be collected. However, inconsistencies in collection protocols—including variations in the time of day, the use of salivary stimulants, and the fasting status of participants—can substantially influence cytokine levels and compromise the reproducibility of assay results. While unstimulated whole saliva is frequently employed, the potential for contamination, such as the presence of blood, may lead to biased outcomes and reduced analytical accuracy [208]. To enhance the reliability and comparability of data across studies, future research should prioritize the development of standardized protocols that define optimal conditions for saliva collection, including consistent fasting status, absence of stimulation, and fixed timing. Additionally, it is essential to establish uniform guidelines for the handling and storage of saliva samples to ensure the biochemical stability of cytokines throughout the analytical process. Equally important is the rigorous validation of assay platforms specifically designed for salivary biomarkers, such as ELISA and multiplex systems, with a focus on their sensitivity and specificity for interleukin detection. These efforts are critical for achieving consistent and reproducible measurements, ultimately paving the way for regulatory acceptance and the broader implementation of salivary diagnostics in routine clinical practice.

### 3.12. Longitudinal Studies and Larger Cohorts

The existing research on salivary biomarkers in psoriasis is frequently constrained by cross-sectional designs and limited sample sizes, which restrict the ability to capture the dynamic nature of the disease, characterized by the alternating periods of remission and exacerbation. Although salivary interleukin levels have the potential to reflect these temporal fluctuations, longitudinal data remain scarce. To advance this field, future investigations should adopt prospective study designs that monitor changes in salivary interleukin concentrations in relation to treatment response, disease relapse, and progression over time. Including diverse patient populations representing a range of disease severities will enhance the external validity and generalizability of findings. Furthermore, establishing correlations between salivary biomarkers and established clinical indices such as the PASI and DLQI is essential for clinical relevance. The retrospective analyses of stored saliva samples from longitudinal cohorts may also provide valuable insights into predictive biomarkers for psoriasis and its associated comorbidities, including psoriatic arthritis [208].

### 3.13. Possibilities of Integration into Clinical Practice

The clinical utility of salivary interleukins relies on their capacity to either complement or potentially replace the existing diagnostic modalities, given that saliva reflects systemic disease activity and is well suited for repeated, non-invasive sampling [162]. Emerging technologies, including lab-on-chip biosensors, are progressing rapidly toward enabling point-of-care cytokine detection, thereby increasing the feasibility of salivary diagnostics in routine clinical settings. Key steps toward clinical integration involve the development of pilot trials aimed at evaluating the feasibility and diagnostic performance of salivary testing in dermatology practices, alongside the comprehensive assessments of cost-effectiveness compared to traditional blood-based assays or imaging techniques. Establishing evidence-based clinical pathways that incorporate salivary interleukin measurements into therapeutic monitoring and decision-making algorithms will be critical. Additionally, widespread implementation will depend on effective patient education and targeted clinician training to ensure the proper utilization and interpretation of salivary biomarker data in clinical practice.

## 4. Conclusions

Salivary interleukins, including IL-1β, IL-6, TNF-α, and IL-17, offer significant results as non-invasive biomarkers for early detection, severity assessment, and treatment monitoring of psoriasis, effectively reflecting systemic inflammation.

The use of saliva as a diagnostic medium offers notable benefits, such as ease of collection, cost-effectiveness, and increased patient compliance.

Incorporating salivary interleukin testing into healthcare technologies could simplify psoriasis management, allowing for timely diagnosis and tailored therapeutic strategies.

Future research should focus on establishing uniform saliva collection guidelines, validating testing platforms, and conducting large-scale studies to define reliable biomarker thresholds.

## Figures and Tables

**Table 1:** Serum cytokine alterations and their clinical significance in psoriasis.

Cytokine	Serum Level in Psoriasis	Correlation with PASI (Disease Severity)	Key Functional Roles
TNF-α	Elevated (in most studies)	Mixed findings; some positive correlation	Major pro-inflammatory cytokine; therapeutic target
IFN-γ	Elevated	Positive correlation	Induces IL-6, IL-8, IL-12, IL-18; lesion formation
IL-6	Elevated	Positive correlation	T cell activation; keratinocyte proliferation; biomarker
IL-8	Elevated	Conflicting results	Neutrophil chemoattractant; erythema promotion
IL-10	Decreased	Not correlated with severity	Anti-inflammatory; immunoregulatory
IL-12	Decreased	Mixed data	Th1 differentiation; IFN-γ production
IL-16	Elevated	Positive correlation	CD4+ lymphocyte recruitment; few studies
IL-17	Mixed results	Inconsistent correlation	Synergizes with IFN-γ; pro-inflammatory role
IL-18	Elevated	Positive correlation	Angiogenesis; immunoregulation; lesion spread
IL-20	Elevated	Positive correlation	Late effector cytokine; keratinocyte dysregulation
IL-22	Elevated	Positive correlation	Barrier defense; disease onset; keratinocyte effects
IL-30	Elevated	Trend toward positive correlation	Modulates Th1/Th17 responses; autoimmune impact

## Data Availability

Not applicable.
